# 

**Published:** 1872-12

**Authors:** 


### INDEX TO VOL. XXVI.

Annual Meeting of the Ohio State Dental Society .............. 31
Anæsthetics .............. 49
Annual Meeting .............. 50
A Visit—Educational .............. 88
A Word for Nitrous Oxide .............. 146
Address by Dr. James Taylor .............. 150
Address by H. A. Smith .............. 233
A case of Adentulous Neuralgia .............. 259
A case of Epulis .............. 260
An Extreme and Successful Case .............. 261
Antidote to Carbolic Acid .............. 268
Address by Dr. E. J. Waye .............. 291
A Substitute for Rubber .............. 363
Answer to Dental Miscellanies .............. 416
A Case .............. 438
A Peculiar Case .............. 448
Amalgam Fillings .............. 457
Aldini Prize on Electricity .............. 490
A Word to Contributors .............. 538
Bone Felon Arrested by Congelation .............. 269
Bibliographical .............. 494
Celluloid Base .............. 94, 148
Communications .............. 9, 53, 97, 141, 233, 277, 321, 363, 407, 451
Correspondence .............. 342, 386, 210
Celluloid .............. 186
California State Dental Association .............. 395
Cure for Sleep-walking .............. 417
Cleaning and Polishing Teeth .............. 466
Cheap Oxygen .............. 298
Connecticut Valley Dental Association .............. 533
Close of the Volume .............. 536
Dental Education .............. 51
Dental Jottings .............. 53, 287
Discussion before the Sixth Annual Meeting of the Ohio State Dental Society, Dec. 5-6, 1871 .............. 122
Discussion in the Mississippi Valley Dental Society .............. 159, 212
Dental Society of New York .............. 180

Dr. Elisha Baker........................................... 181
Departures—Professional.................................... 185
Dental Miscellanies.........................................334, 458
Dental Teaching............................................ 337
Diamond Drills............................................. 363
Dentists and Dental Students............................... 377
Difficulties in Dental Practice............................ 405
Dental Periostitis.......................................... 418
Deterioration of Pulpless Teeth............................ 447
Disease and the Weather.................................... 488
Death from Chloroform...................................... 489
Dental Law................................................. 491
Dr. Hurd................................................... 230
Dental Chemistry........................................... 495
Dental Pathology and Surgery............................... 517
Dentistry—Its Difficulties and Responsibilities............ 508
Editorial..................51, 88, 136, 185, 273, 318, 353, 404, 447, 491
Eastern Ohio Dental Association.............................436, 133
Extracting Teeth under the Influence of Anæsthetics in the Lying Down Posture.............................................. 373
Expression of Appreciation................................. 449
Ether and Chloroform....................................... 470
Editorial Experiences...................................... 537
Filling Teeth.............................................. 106
Filters and Filtering...................................... 317
Fine Art in Dentistry...................................... 324
From Europe................................................ 386
From Florence, Italy....................................... 523
Green's Improved Burr Engine............................... 183
Glycerine as a Solvent for Hypodermic Injections........... 269
Gold, Gold, Gold........................................... 450
Hypophosphites in Toothache................................ 268
Horace Wells Testimonial Fund.............................. 404
Indiana State Dental Society................................270, 426
Illinois State Dental Society.............................. 299
Is the Dentine Tubular..................................... 363
Iowa Dental Society........................................ 437
Interdentinal Splint—With Case in Practice................. 198
Inflammation............................................... 228
Kansas and Missouri Valley Dental Association.............. 343
Kentucky State Dental Association.......................... 357
Local Variations of Disease................................ 257
Let History be Accurate.................................... 342
Latent Senses.............................................. 480
Mechanical Dentistry as an Art—Its Decline................. 19

Management of a Difficult Case . . . . . 30
Monsieur Tonson Come Again—Rubber Subject . . . . . 92
Mississippi Valley Dental Society . . . . . 95, 217
Mississippi Valley Dental Meeting . . . . . 137
Mass Convention of the Dentists of the West . . . . . 139
Meeting of the Eastern Dental Association . . . . . 184
M. D. and D. D. S . . . . . 284
Meeting of the Northern Ohio Dental Society . . . . . 346
Meeting of the Central Michigan Dental Association . . . . . 357
Mad River Valley Dental Society . . . . . 383, 433
Mad River Valley Dental Society . . . . . 433
Michigan State Dental Association . . . . . 471
Neuralgia . . . . . 118
Northern Ohio Dental Society . . . . . 275
Notes on Hospital Practice . . . . . 486
Ohio State Dental Society—Address of Dr. George W. Keely, retiring President . . . . . 23
Old Colony Dental Society . . . . . 88
Ohio Dental College Association . . . . . 94
Office Notes—Destroying Nerves . . . . . 115
Ohio Dental College . . . . . 136, 406
Ohio College of Dental Surgery . . . . . 138
Offensive Breath . . . . . 256
Ohio Valley Dental Association . . . . . 363
Ossified Pulps . . . . . 367
On Idiosyncrasies . . . . . 439
Ohio State Dental Society . . . . . 491
Obscure Case . . . . . 494
Oxychloride of Zinc . . . . . 515
Our Profession . . . . . 535
Prof. Wright and Operative Dentistry . . . . . 27
Poisoned by Mercury from a Filling . . . . . 47, 145, 189
Pass Him Around . . . . . 345
Ptyalism from Amalgam Fillings . . . . . 384
Phosphate of Lime—Suggestions as to its Use by Dentists . . . . . 412
Proceedings of the Ninth Annual Meeting of the Tennessee Dental Association . . . . . 525
Running the Risk . . . . . 97
Reasonable Talk on the Subject of Filling Teeth . . . . . 200
Report on Filling Teeth . . . . . 463
R. W. Varney . . . . . 231
Report on Dental Literature . . . . . 520
Safe Anæsthesia . . . . . 48
Still at School . . . . . 91
Severe Ptyalism from a Dental Operation without Amalgam . . . . . 141

Soft Foil vs. Adhesive Foil.......... 142
Spring Course.......... 187
Second District Dental Society of New York.......... 219
Suggestions in Reference to the Cummings Patent.......... 271
State Board of Dental Examiners..........275, 491
Structure and Development of the Teeth.......... 277
Screws.......... 406
Syrup of the Lacto-Phosphate of Lime.......... 445
Sensitive Dentine.......... 499
The Extraction of Roots—When is it Indicated?.......... 9
The Dentists—Kentucky State Dental Association—How it was Organized, by Whom, and for What Purpose.......... 42
Two Usefnl Things.......... 120
The Annual Meeting of the Lake Erie Dental Association.......... 184
The State Board of Examiners.......... 187
Teeth Fibrous.......... 194
The Vehicle for Chloral.......... 209
The Treatment of Inflammation.......... 221
The Rubber Patent Case.......... 230
The Departed.......... 231
Tribute of Respect.......... 232
Treatment of Irregularity.......... 243
The Supply of Lime Phosphates to the Mother during Periods of Gestation and Lactation.......... 246
Tannin and Glycerine.......... 255
The Cummings Patent.......... 318
The American Dental Association..........,320, 361
The Period When Teeth Begin to Form.......... 321
Thoughts on Success in Dental Practice.......... 329
Thymol.......... 360
The Nerves of Taste.......... 383
The Dentine Cellular and Fibrous.......... 407
Toothache.......... 422
To Prevent Stains from Tincture of Iodine.......... 446
The Object of Our Profession.......... 464
Tumor in Mammary Gland.......... 483
Tennessee State Dental Society.......... 492
The Action of Creosote and Carbolic Acid.......... 505
Unnecessary and Unkind.......... 206
Valedictory Address to the Graduating Class of Belleview Medical College.......... 45
What will be the Result?.......... 230
What is our Duty to Those Who Enter our Offices as Students, Relative to our Profession?.......... 250
What Shall be Done with Josiah?.......... 361
What Will Make Good Teeth?.......... 451

AMERICAN DENTAL ASSOCIATION—Meets on the first Tuesday in August next year at Niagara Falls. *Pres.* GEO. W. CUSHING, Chicago, Ills. *Rec. Sec.* M. S. DEAN, Chicago.

AMERICAN DENTAL CONVENTION.—Meets in Saratoga, Aug. 9, 1871. *Pres.* Dr. J. G. AMBLER. *Sec.* Dr. J. S. LATIMER, New Haven, Conn.

*List of Societies represented in the American Dental Association.*

AMERICAN ACADEMY OF DENTAL SCIENCE—Meets at Boston, Mass. *Pres.* DANIEL HARWOOD, M. D., of Boston. *Sec.* L. D. SHEPARD, D. D. S., of Boston.

BROOKLYN DENTAL SOCIETY—Meets semi-monthly. *Pres.* Dr. A. H. Brockway. *Rec Sec.* J. C. Wyman. *Cor. Sec.* C. A. Marvin.

BUFFALO DENTAL SOCIETY—Meets monthly at Buffalo. *Pres.* B. T. Whitney, *Rec. Sec.* S. A. FREEMAN.

BOSTON DENTAL INSTITUTE. Meets first Tuesday of each month. *Pres.* D. G. Williams. *Cor. Sec.* D. S. Dickerman.

CALIFORNIA STATE DENTAL ASSOCIATION.—Meets annually in June. *Pres.* S. W. DENNIS, San Francisco, *Rec. Sec.* H. J PLOMTEAUX.

CINCINNATI DENTAL SOCIETY—Meets semi-monthly at Cincinnati. *Pres.* Geo. WATT, *Rec. Sec.* Wm. TAFT.

CHICAGO DENTAL SOCIETY—Meets monthly at Chicago. *Pres.* GEO. H. CUSHING. *Rec and Cor. Sec.* EDGAR D. SWAIN.

CONNECTICUT VALLEY DENTAL ASSOCIATION—Meets semi-annually second Tuesday of May and October. *Pres.* A. A. HOWLAND, Barre, Vt. *Rec. Sec.* W. H. JONES, of Boston, Mass.

CONNECTICUT STATE DENTAL ASSOCIATION—*Pres.* H. L. SAGE, Bridgeport. *Cor. Sec.* JOHN CODY, Hartford. *Rec. Sec.* C. A POWERS, Hartford.

CHARLESTON DENTAL ASSOCIATION.*—*Prest.* I. B. PATRICK. *Sec. and Treas.* T. F. CHUPEIN.

DELAWARE DENTAL ASSOCIATION—Meets semi-annually. *Pres.* E. W. HAINES, Newark. *Sec.* C. R. JEFFRIES, Wilmington.

EASTERN OHIO DENTAL ASSOCIATION—Meets on Tuesday, Februery 27th, 1872, at Alliance. *Pres.* J. C. WHINERY *Sec.* D. B. MCLAIN.

EAST TENNESSEE DENTAL ASSOCIATION—Meets annually at Knoxville. Tenn *Pres.* WM. H. COOKE, of Cleveland. *Rec. Sec.* A. P. WHITE, Knoxville.

FOREST CITY SOCIETY OF DENTAL SURGEONS. *Pres.* B. Strickland, Cleveland. *Rec. Sec.*, H. L. Ambler, Cleveland. *Cor. Sec.* Chas. Buffett, Cleveland.

GEORGIA STATE DENTAL SOCIETY—Next meeting at Atlanta, July 30th, 1870. *Pres.* F. Y. CLARK; *Cor. Sec.* J. P. H. BROWN, Augusta.

HUDSON RIVER ASSOCIATION OF DENTAL SURGEONS—Meets on the second Wednesdays of May, September and January. *Pres.* L. S. STRAW, Newburg N. Y. *Rec. Sec.* T. W. DUBOIS, Pougkeepsie, N. Y.

HUDSON VALLEY DENTAL ASSOCIATION—meets monthly at Troy, N. Y. *Pres.* L. C WHEELER, *Rec. Sec.* S. G. ANDRES, *Cor. Sec.* S. D. FRENCH.

ILLINOIS STATE DENTAL SOCIETY—Meets annually. *Pres.* O. Wilson, of Aurora. *Sec.* C. S. SMITH, of Springfield. Next meeting second Tuesday of May, 1872, at Chicago

INDIANA STATE DENTAL SOCIETY—Meets annually at Indianapolis on the last Tuesday of June. *Pres* P. G. C. HUNT, of Indianapolis. *Rec. and Cor. Sec* S. B. Brown

IOWA STATE DENTAL SOCIETY—Meets annually. *Next meeting at Iowa City, on the second Tuesday of July. *Pres.* J. HARDMAN, of Muscatine. *Rec. Sec.* A. B MASON, of Waterloo. *Cor. Sec.* J. P. WILSON, of Burlington.

KANSAS STATE DENTAL SOCIETY—Meets semi-annually. Next meeting at Topeka Sept. 12, 1871, the next at Atchison, May 2, 1872. *Pres.* J. B. WHEELER. *Sec.* E. C. FULLER

KENTUCKY STATE DENTAL ASSOCIATION—Meets annually, and Semi monthly, in the City of Louisville. Annual meeting, First Tuesday in June, at 2 o'clock P. M. Semi-monthly, meetings 2d and 4th Tuesday Nights. W. G. REDMAN, *Pres't.* W. H. SHADOAN *Sec.* Both at Louisville,

LAKE ERIE DENTAL ASSOCIATION.—Meets Annually.* *Pres.* J. G. TEMPLETON. *Sec.* C. B. ANSART. Next meeting first Tuesday of May, 1872, at Conneautville, Pa.

MAD RIVER VALLEY DENTAL SOCIETY—Meets semi annually on the 1st Tuesday in May next at Yellow Springs, O. *Pres.* A. A. BLOUNT, Springfield, O. *Sec.* R. H. BOAL, Urbana, O.

MAINE DENTAL SOCIETY—Meets in August, prox., at Biddeford. *Pres.* J. B. FILLEBRONN, *Sec.* E. J. ROBERTS, of Augusta.

MARYLAND ASSOCIATION OF DENTISTS—Meets the last Thursday of every month. *Pres.* J. B. BEAN, *Sec.*, S. M. FIELD.

MASSACHUSETTS DENTAL SOCIETY—Meets monthly. *Pres.* A. A. Cook. *Rec. Sec.* C. Wilson, *Cor. See.* L. D. Shepard

MICHIGAN STATE DENTAL ASOCIATION—Meets annually 2d Tuesd y of Oct at Gr. Rapids, *Pres.* E. S. HOLMES, of Grand Rapids; *Rec. and Cor. Sec.* G. P. HOLMES, Battle Creek

MICHIGAN CENTRAL DENTAL ASSOCIATION—Meets annually. *Pres.* D. C. HAWXHURST. *Sec.* H. P. PETERSON. Meets at Jackson, second Wednesday of January, 1872.

MISSISSIPPI VALLEY DENTAL ASSOCIATION—Meets annually at Cincinnati, on the first Wednesday in March. *Pres.* H. A. SMITH, Cincinnati. *Rec. Sec.* C. M. WRIGHT Cinicinnati, O.

MISSOURI DENTAL ASSOCIATION—Meets on the first Tuesday of June, in S Louis. *Pres.* H. JUDD, St. Louis, *Sec.* W. H. EAMES, St. Louis.

MISSOURI VALLEY DENTAL SOCIETY.* Meets on the second Tuesday in July and January. *Pres.* E. J. WOODBURY of Council Bluffs, Iowa *Sec. and Treas.* J. F. Sanborn, Tabor, Iowa.

MERRIMAC VALLEY DENTAL SOCIETY—Meets semi annually first Tuesday of Oct. and May.* *Pres.* I. H. KIDDER, Mass. *Rec. Sec.* A. M. DUDLEY, of Lowell *Cor. Sec.* D. T. PORTER, Lawrence, Mass.

MEMPHIS DENTAL SOCIETY.—Meets on the first Tuesday of each month. *Pres* W. T. Arrington, *Sec.* V. F. Elliott, *Treas.* C. L. Harris.

NASHVILLE DENTAL ASSOCIATION—Meets on the second Tuesday of each month *Pres.* J C. ROSS, *Sec* R. R. FREEMAN, *Cor. Sec.* S. J. COBB.

NEW JERSEY STATE DENTAL SOCIETY.—Meets annually. Next meeting at Long Branch, on the second Tuesday in July, 1871. *Pres.* A. W. KINGSLEY of Elizabeth, *Sec.* E. F. HANKS of Rahway.

NEW YORK STATE DENTAL SOCIETY—Meets at Albany on the last Tuesday of June next *Pres.* L W ROGERS, of Utica. *Sec.* CHAS. BARNS, of Syracuse *Cor. Sec.* W. H. ATKINSON, New York. *State Censors*, S. H. MCCALL, Binghamton, 6th Dist. R. G. SNOW, Buffalo, 8th Dist.

NEW HAVEN DENTAL SOCIETY—Meets on the first Wednesday of each month New Haven. *Pres.* J. T. METCALF, *Rec. and Cor. Sec.* C. L. SMITH, of New Haven.

NORTHERN OHIO DENTAL ASSOCIATION—Meets annually, (last Tuesday of May.) at Put in Bay. *Pres.* E. J. WAY, Sandusky; *Rec. Sec.* H H. NEWTON, Cleveland.

NORTHERN IOWA DENTAL ASSOCIATION—Meets annually, on the 2d Tuesday in June, 1870. Next meeting at Waterloo. *Pres.* J. T. ABBOTT, Manchester, *Rec. Sec* A. V. EATON, Anamosa. *Cor. Sec.* A B. MASON, Waterloo.

OHIO DENTAL COLLEGE ASSOCIATION—Meets annually in March, at Cincinnat. *Pres.* W. H. GODDARD, Louisville, Ky. *Rec Sec.* J. TAFT, of Cincinnati.

OHIO STATE DENTAL ASSOCIATION.—Meets annually, at Columbus. [Next meeting—first Wednesday of December.] *Pres.* B. T. SPELLMAN, Warren. *Rec. Sec.* C. R. TAFT, Mansfield, O. *Cor. Sec.* C. R. BUTLER, Cleveland, O.

ODONTOGRAPHIC SOCIETY OF PENNSYLVANIA—Meets monthly in Philadelphia Next meeting, Wednesday, Sept 1st. *Pres.* J. H. MCQUILLEN. *Rec. Sec.* A. BOICE. *Cor. Sec.* T. C. STELLWAGEN.

ODONTOLOGICAL SOCIETY OF NEW YORK CITY—Meets the second Tuesday of each month.* *Pres.* C. E. FRANCIS *Rec Sec.* C. F. IVES.

OLD COLONY DENTAL ASSOCIATION—Meets quarterly.* *Pres.* C. G. Davis *Rec. Sec.* L. W. PUFFER, North Bridgewater, Mass. *Cor. Sec.* N. C. FOWLER, Yarmouth, Mass.

PENNSYLVANIA ASSOCIATION OF DENTAL SURGEONS—Meets monthly in Phil. *Pres.* J. T. TEMPLETON. *Rec. Sec* C. H. BAGLEY, Meadville *Cor. Sec.* S. WELCHENS.

SAN FRANCISCO DENTAL ASSOCIATION.—Meets monthly. *Pres.* HENRY AUSTIN *Cor. Sec.* H. E. KNOX *Rec. Sec.* A. B. WOOD.

POUGHKEEPSIE DENTAL SOCIETY.—Meets monthly, *Pres.* J. H. Mann, *Sec.* H. F Clark.

ST. LOUIS DENTAL SOCIETY—Meets monthly. *Pres.* ISAAC COMSTOCK. *Sec.* R. J. POORE

SUSQUEHANNA DENTAL ASSOCIATION—Meets annually*. *Pres.* J. D. WINGATE, *Rec. Sec.* J. M. BARRETT.

SOCIETY OF DENTAL SURGEONS OF THE CITY OF NEW YORK—Meets semi monthly in New York. *Pres.* B. W FRANKLIN. *Rec. Sec.* J. S. LATIMER. *Cor. Sec.* T. H. BURRAS.

SOUTEHRN DENTAL ASSOCIATION.—Meets annually.* Next meeting the 2d Wednesday in April, 1870, at Charleston, S. C. *Pres.* JAS. KNAPP. *Rec. Sec.* Prof. J. G. ANGELL, New Orleans.

SOUTH CAROLINA STATE DENTAL ASSOCIATION.—Meets semi-annually in May and Nov. *Pres.* W. C. WARDLAW Abbeville; *Sec.* O. J. BOND, Marion.

STATE DENTAL SOCIETY OF PENNSYLVANIA.—Meets annually, First Tuesday in June. Next meeting in Erie, 1872. *Pres.* J. G. TEMPLETON, New Castle. *Rec Sec.* C. H. BAGLEY, Meadville. *Cor. Sec.* SAMUEL WELCHENS, Lancaster.

TENNESSEE DENTAL ASSOCIATION.—Meets semi-annually. *Pres.* W. T. Arrington of Memphis, *Sec.* —— of Somerville.

WASHINGTON CITY DENTAL SOCIETY—Meets monthly. *Pres.* R. F. HUNT. *Sec.* Dr. EVANS.

WABASH VALLEY DENTAL ASSOCIATION.—Meets annually. Next meeting at Fort Wayne third Tuesday in March, 1872. *Pres.* A. B. CUNNINGHAM, Attica; *Sce.* S. B. BROWN, Fort Wayne.

WISCONSIN STATE DENTAL ASSOCIATION—Meets semi-annually.* Next meeting at Watertown, second Tuesday of January, 1872. *Pres* EDGAR PALMER, La Crosse, *Sec.* C C. CHITTENDEN, Madison.

***Place of meeting fixed by appointment.**

### DTRICT DENTAL SOCIETIES OF THE STATE OF NEW YORK.

*1ST DISTRICT DENTAL SOCIETY.—*Pres.* W. H. Atkinson, New York City. *Sec.* O. A. JARVIS, New York City.

2D DISTRICT DENTAL SOCIETY.—*Pres.* W. B. Hurd, Brooklyn. *Rec. Sec.* Wm. Jarvie Jr., Brooklyn. *Cor. Sec.* L S. Straw, Newburg

3D DISTRICT DENTAL SOCIETY.—Meets at Albany semi-annually and annually. *Pre-* J. A. Perkins, Troy. *Sec.* S. J. Andress, of Albany.

*4TH DISTRICT DENTAL SOCIETY.—*Pres.* F. C. Rich, Saratoga Springs. *Sec.* C. A Elmore, Fort Edwards.

*5TH DISTRICT DENTAL SOCIETY.—*Pres.* G. A. Foster, Utica. *Sec.* Charles Barnes Syracuse.

*6TH DISTRICT DENTAL SOCIETY.—*Pres* A. M. Holmes. *Sec.* C. A. Perkins. Semi annual meeting at Ithaca, Sept. 26, 1871.

*7TH DISTRICT DENTAL SOCIETY.—*Pres.* A. G. COLEMAN, Canandagua. *Sec.* H. S MILLER, Rochester.

8TH DISTRICT D NTAL SOCIETY.—Meets annually on the 1st Tuesday in May, as Buffalo. *Pres.* W. C. BARRETT. *Sec.* S A. FREEMAN, Buffalo.

THE 7TH AND 8TH DISTRICT SOCIETIES—Hold a union meeting on the first Tuesday of October, alternately at Buffalo and Rochester.

* Place and time of meeting fixed by appointment.

The semi-annual meeting of the Ohio State Board of Dental Examiners will be held in Cincinnati, on Wednesday, June 5th, 1872, at 10 o'clock A. M.

J. TAFT, President.

W. P. HORTON, Secretary.

### EXCHANGES.

ST. LOUIS MEDICAL AND SURGICAL JOURNAL,
BOSTON MEDICAL AND SURGICAL JOURNAL,
THE PACIFIC MEDICAL AND SURGICAL JOURNAL, SAN FRANCISCO.
BUFFALO MEDICAL AND SURGICAL JOURNAL,
PHILADELPHIA MEDICAL AND SURGICAL REPORTER,
CINCINNATI LANCET AND OBSERVER,
DENTAL COSMOS, PHILADELPHIA.
BRAITHWAITE'S RETROSPECT, N. Y.
THE DENTAL TIMES, PHILADELPHIA,
LADIES' REPOSITORY, CINCINNATI.
HALL'S JOURNAL OF HEALTH, N. Y.
CHICAGO MEDICAL EXAMINER.
THE DETROIT REVIEW OF MEDICINE AND PHARMACY.
MEDICAL RECORD, N Y.
NASHVILLE JOURNAL OF MEDICINE AND SURGERY,
AMERICAN ECLECTIC MEDICAL REVIEW, N. Y.
AMERICAN JOURNAL OF DENTAL SCIENCE, BALTIMORE.
THE UNITED STATES MEDICAL AND SURGICAL JOURNAL, CHICAGO,
NEW ORLEANS JOURNAL OF MEDICINE.
WESTERN JOURNAL OF MEDICINE. INDIANAPOLIS, IND.
AMERICAN AGRICULTURIST, N. Y.
DENTAL OFFICE AND LABORATORY, PHILA.
BOSTON JOURNAL OF CHEMISTRY.
CINCINNATI MEDICAL REPERTORY.
JOURNAL OF APPLIED CHEMISTRY. N. Y.
DRUGGISTS' CIRCULAR AND CHEMICAL GAZETTE, NEW YORK.
CANADA JOURNAL OF DENTAL SCIENCE, MONTREAL.
OHIO CONVENTION REPORTER, COLUMBUS.
INDIANA JOURNAL OF MEDICINE.
JOURNAL OF THE GYNÆCOLOGICAL SOCIETY, BOSTON.
MEDICAL AND SURGICAL REPORTER, SALEM, OREGON.
WOOD'S HOUSEHOLD MAGAZINE, NEWBURG, N. Y.
MISSOURI DENTAL JOURNAL, ST. LOUIS.
ECLECTIC MEDICAL JOURNAL, CINCINNATI.
INDUSTRIAL PRESS, CINCINNATI.
TEMPERANCE ALBUM, NORTH ADAMS, MASS.

### New Orleans Dental College

*Cor. of Carondelet & Perdido Sts.*

---

### FOURTH SESSION—1870-71.

---

### *FACULTY.*

JAS. S. KNAPP, D. D. S.,
Professor of Theory and Practice.

ANDREW F. McLAIN, D. D. S., M. D.,
Professor of Institutes of Dentistry.

WM. S. CHANDLER, D. D. S.,
Professor and Clinical Instructor of Dental Surgery.

CHAS. E. KELLS, D. D. S.,
Professor of the Science of Dental Mechanism.

S. MINTER ANGELL, M. D.,
Professor of Anatomy and Physiology.

J. S. HARRISSON, A. M, M. D.,
Professor of Materia and Medical Jurisprudence.

JOHN G. ANGELL, D. D. S.,
Clinical Instructor of Dental Mechanism and
Adjunct Prof. Operative Dental Surgery.

S. P. CUTLER, M. D., D. D. S.,
Prof. of Chemistry, Metallurgy, Microscopy and Histology.

---

DEAN OF THE FACULTY, - - - - - JAMES S. KNAPP, D. D. S

*Office, No. 15 Baronne Street, New Orleans.*

---

The Fourth Regular Course of Lectures of the New Orleans Dental College for the Session of 1870-71. will commence on the 15th of November and continue four months.

The superior advantages of this location for a Dental College should not be forgotten by students who live in a colder climate, and who by coming here will avoid the severity and rigors of a Northern winter, and enjoy four months of comparatively mild and pleasant weather. To those in delicate health this is an inducement of no inconsiderable importance.

| | |
|---|---|
| **Professor's Tickets, for full course,** | **$100 00** |
| **Matriculation (paid once),** | **5 00** |
| **Diploma Fee,** | **30 00** |

Board and lodging can be procured as cheaply in New Orleans as any large city in this country.

For further information address the Dean of the Faculty,

J. S. KNAPP, D. D. S.
Office No. 15 Baronne St., New Orleans

Or the Secretary,

PROF. A. F. McLAIN, D. D. S., M. D.
Acting Dean. Box 83,

New Orleans, May 21, 1870. May, '70.

### OHIO COLLEGE
OF
### DENTAL SURGERY.

The Twenty-sixth Annual Session of the Ohio College of Dental Surgery will commence on Tuesday the 17th of October next, and continue until March 1st, '72.

### FACULTY.

JAMES TAYLOR, M. D., D. D. S.,
Emeritus Professor of Institutes of Dental Science.

C. KEARNS, M. D.,
Professor of Anatomy.

D. VAUGHAN,
Professor of Chemistry and Therapeutics.

EDWARD RIVES, M. D.,
Professor of Physiology and Histology.

F. BRUNNING M. D.,
Professor of Pathology and Therapeutics.

J. TAFT, D. D. S.,
Professor of Operative Dentistry and Dental Hygiene.

C. M. WRIGHT, D. D. S.,
Professor of Mechanical Dentistry and Metallurgy.

WILL TAFT, D. D. S.,
Professor of Clinical Dentistry.

A. SCHWAGMEYER, M. D.
Demonstrator of Anatomy.

### TEXT BOOKS.

Gray's and Wilson's Anatomy; Dalton's or Carpenter's Physiology; Williams' Pathology; Fown's, Turner's, Graham's, or Brandt and Taylor's Chemistry; Taft's Operative Dentistry; Richardson's Mechanical Dentistry; Harris' Principles and Practice of Dental Science, Tomes Dental Surgery, Watts' Chemical Essays, Harris' Dental Dictionary.

### TERMS OF ADMISSION.

Tickets must be procured at the beginning of the session.

| | |
|---|---|
| Tickets for the course, | $100 00 |
| Demonstrator's Ticket for Anatomy, | 5 00 |
| Matriculation Fee, but once, | 5 00 |
| Diploma Fee, | 30 00 |

### TERMS OF GRADUATION.

Two courses, (including one of dissections,) with a satisfactory dental pupilage.

Eight years reputable practice, or a satisfactory examination upon the branches of the Junior Course in this Institution will be received as equal to one course.

An acceptable thesis upon some subject on Dental science.

Must possess a good moral character, and be twenty-one years old.

For further information, address J. TAFT, *Dean.*
117 West Fourth Street.

### New Orleans Dental College

*Cor. of Carondelet & Perdido Sts.*

---

### FOURTH SESSION—1870-71.

---

### *FACULTY.*

JAS. S. KNAPP, D. D. S.,
Professor of Theory and Practice.

ANDREW F. McLAIN, D. D. S., M. D.,
Professor of Institutes of Dentistry.

WM. S. CHANDLER, D. D. S.,
Professor and Clinical Instructor of Dental Surgery.

CHAS. E. KELLS, D. D. S.,
Professor of the Science of Dental Mechanism.

S. MINTER ANGELL, M. D.,
Professor of Anatomy and Physiology.

J. S. HARRISSON, A. M., M. D.,
Professor of Materia and Medical Jurisprudence.

JOHN G. ANGELL, D. D. S.,
Clinical Instructor of Dental Mechanism and
Adjunct Prof. Operative Dental Surgery.

S. P. CUTLER, M. D., D. D. S.,
Prof. of Chemistry, Metallurgy, Microscopy and Histology.

---

DEAN OF THE FACULTY, - - - - JAMES S. KNAPP, D. D. S

*Office, No. 15 Baronne Street, New Orleans.*

---

The Fourth Regular Course of Lectures of the New Orleans Dental College for the Session of 1870-71 will commence on the 15th of November and continue four months.

The superior advantages of this location for a Dental College should not be forgotten by students who live in a colder climate, and who by coming here will avoid the severity and rigors of a Northern winter, and enjoy four months of comparatively mild and pleasant weather. To those in delicate health this is an inducement of no inconsiderable importance.

| | |
|---|---|
| **Professor's Tickets, for full course,** - - - - - - | **$100 00** |
| **Matriculation (paid once),** - - - - - - - - - - - - - | **5 00** |
| **Diploma Fee,** - - - - - - - - - - - - - - - - - - | **30 00** |

Board and lodging can be procured as cheaply in New Orleans as any large city in this country.

For further information address the Dean of the Faculty,

J. S. KNAPP, D. D. S.
Office No. 15 Baronne St., New Orleans

Or the Secretary,

PROF. A. F. McLAIN, D. D. S., M. D.
Acting Dean. Box 83½

New Orleans, May 21, 1870. May, '70.

### New Orleans Dental College

***Cor. of Carondelet & Perdido Sts.***

### FOURTH SESSION—1870-71.

### *FACULTY.*

JAS. S. KNAPP, D. D. S.,
Professor of Theory and Practice.

ANDREW F. McLAIN, D. D. S., M. D.,
Professor of Institutes of Dentistry.

WM. S. CHANDLER, D. D. S.,
Professor and Clinical Instructor of Dental Surgery

CHAS. E. KELLS, D. D. S.,
Professor of the Science of Dental Mechanism.

S. MINTER ANGELL, M. D.,
Professor of Anatomy and Physiology.

J. S. HARRISSON, A. M., M. D.,
Professor of Materia and Medical Jurisprudence.

JOHN G. ANGELL, D. D. S.,
Clinical Instructor of Dental Mechanism and
Adjunct Prof. Operative Dental Surgery.

S. P. CUTLER, M. D., D. D. S.,
Prof. of Chemistry, Metallurgy, Microscopy and Histology.

Dean of the Faculty, - - - - JAMES S. KNAPP, D. D. S

*Office, No. 15 Baronne Street, New Orleans.*

The Fourth Regular Course of Lectures of the New Orleans Dental College for the Session of 1870-71, will commence on the 15th of November, and continue four months.

The superior advantages of this location for a Dental College should not be forgotten by students who live in a colder climate, and who by coming here will avoid the severity and rigors of a Northern winter, and enjoy four months of comparatively mild and pleasant weather. To those in delicate health this is an inducement of no inconsiderable importance.

**Professor's Tickets, for full course,** - - - - - - - $100 00
**Matriculation (paid once),** - - - - - - - - - - - - - 5 00
**Diploma Fee,** - - - - - - - - - - - - - - - - - - 30 00

Board and lodging can be procured as cheaply in New Orleans as any large city in this country.

For further information address the Dean of the Faculty,

J. S. KNAPP, D. D. S,
Office No. 15 Baronne St., New Orleans

Or the Secretary,

PROF. A. F. McLAIN, D. D. S., M. D.

Acting Dean, Box 83[illegible]

New Orleans, May 21, 1870. May, '70.

### OHIO COLLEGE
OF
### DENTAL SURGERY.

The Twenty-sixth Annual Session of the Ohio College of Dental Surgery will commence on Tuesday the 17th of October next, and continue until March 1st, '72.

### FACULTY.

JAMES TAYLOR, M. D., D. D. S.,
Emeritus Professor of Institutes of Dental Science.

C. KEARNS, M. D.,
Professor of Anatomy.

D. VAUGHAN,
Professor of Chemistry and Therapeutics.

EDWARD RIVES, M. D.,
Professor of Physiology and Histology.

F. BRUNNING M. D.,
Professor of Pathology and Therapeutics.

J. TAFT, D. D. S.,
Professor of Operative Dentistry and Dental Hygiene

C. M. WRIGHT, D. D. S.,
Professor of Mechanical Dentistry and Metallurgy.

WILL TAFT, D. D. S.,
Professor of Clinical Dentistry.

A. SCHWAGMEYER, M. D.
Demonstrator of Anatomy.

### TEXT BOOKS.

Gray's and Wilson's Anatomy; Dalton's or Carpenter's Physiology; Williams' Pathology; Fown's, Turner's, Graham's, or Brandt and Taylor's Chemistry; Taft's Operative Dentistry; Richardson's Mechanical Dentistry; Harris' Principles and Practice of Dental Science, Tomes Dental Surgery, Watts' Chemical Essays, Harris' Dental Dictionary.

### TERMS OF ADMISSION.

Tickets must be procured at the beginning of the session.

| | |
|---|---|
| Tickets for the course, | $100 00 |
| Demonstrator's Ticket for Anatomy, | 5 00 |
| Matriculation Fee, but once, | 5 00 |
| Diploma Fee, | 30 00 |

### TERMS OF GRADUATION.

Two courses, (including one of dissections,) with a satisfactory dental pupilage.

Eight years reputable practice, or a satisfactory examination upon the branches of the Junior Course in this Institution will be received as equal to one course.

An acceptable thesis upon some subject on Dental science.

Must possess a good moral character, and be twenty-one years old.

For further information, address J. TAFT, *Dean.*
117 West Fourth Street.

### OHIO COLLEGE

OF

### DENTAL SURGERY.

The Twenty-sixth Annual Session of the Ohio College of Dental Surgery will commence on Tuesday the 17th of October next, and continue until March 1st, '72.

### FACULTY.

JAMES TAYLOR, M. D., D. D. S.,
Emeritus Professor of Institutes of Dental Science.

C. KEARNS, M. D.,
Professor of Anatomy.

D. VAUGHAN,
Professor of Chemistry and Therapeutics.

EDWARD RIVES, M. D.,
Professor of Physiology and Histology.

F. BRUNNING M. D.,
Professor of Pathology and Therapeutics.

J. TAFT, D. D. S,
Professor of Operative Dentistry and Dental Hygiene

C. M. WRIGHT, D. D. S,
Professor of Mechanical Dentistry and Metallurgy,

WILL TAFT, D. D. S.,
Professor of Clinical Dentistry.

A. SCHWAGMEYER, M. D.
Demonstrator of Anatomy.

### TEXT BOOKS.

Gray's and Wilson's Anatomy; Dalton's or Carpenter's Physiology; Williams' Pathology; Fown's, Turner's, Graham's, or Brandt and Taylor's Chemistry; Taft's Operative Dentistry; Richardson's Mechanical Dentistry; Harris' Principles and Practice of Dental Science, Tomes Dental Surgery, Watts' Chemical Essays, Harris' Dental Dictionary.

### TERMS OF ADMISSION.

Tickets must be procured at the beginning of the session.

| | |
|---|---|
| Tickets for the course, | $100 00 |
| Demonstrator's Ticket for Anatomy, | 5 00 |
| Matriculation Fee, but once, | 5 00 |
| Diploma Fee, | 30 00 |

### TERMS OF GRADUATION.

Two courses, (including one of dissections,) with a satisfactory dental pupilage.

Eight years reputable practice, or a satisfactory examination upon the branches of the Junior Course in this Institution will be received as equal to one course.

An acceptable thesis upon some subject on Dental science.

Must possess a good moral character, and be twenty-one years old.

**For further information, address** J. TAFT, *Dean.*
**117 West Fourth Street.**

### NEW ORLEANS
### DENTAL COLLEGE,

### Cor. Carondelet and Perdido Streets.

---

### SIXTH SESSION--1872-73.

---

### FACULTY:

JAS. S. KNAPP, D. D. S.,
Professor of Theory and Practice.
ANDREW F. McLAIN, D. D.S., M. D.,
Professor of Institutes of Dentistry.
WM. S. CHANDLER, D. D. S.,
Professor and Clinical Instructor of Dental Surgery.
CHAS. E. KELLS, D. D. S.,
Professor of the Science of Dental Mechanism.
S. MINTER ANGELL, M. D.,
Professor of Anatomy and Physiology.
J. S. HARRISSON, A. M., M. D.
Professor of Materia and Medical Jurisprudence.
JOHN G. ANGELL, D. D. S.,
Clinical Instructor of Dental Mechanism and Adjunct Prof. Operative Dental Surgery.
S. P. CUTLER, M. D., D. D. S.,
Prof. of Chemistry, Metallurgy, Microscopy and Histology.

---

DEAN OF THE FACULTY, - - - JAMES S. KNAPP, D. D. S.

*Office, No.* 15 *Buronne Street, New Orleans.*

---

The Fourth Regular Course of Lectures of the New Orleans Dental College for the Session of 1872-73, will commence on the 15th of November, and continue four months. The superior advantages of this location for a Dental College should not be forgotten by students who live in a colder climate, and who by coming here will avoid the severity and rigors of a Northern winter, and enjoy four months of comparatively mild and pleasant weather. To those in delicate health this is an inducement of no inconsiderable importance.

| | |
|---|---|
| **Professor's Tickets for full course, - - - - - -** | **$100 00** |
| **Martriculation (paid once), - - - - - - - - -** | **5 00** |
| **Diploma Fee, - - - - - - - - - - - - - - - - -** | **30 00** |

Board and lodging can be procured as cheaply in New Orleans as any large city in this country.

For further information address the Dean of the Faculty,

J. S. KNAPP, D. D. S.
Office No. 15 Baronne St., New Orleans,

Or the Secretary,
PROF. A. F. McLAIN, D. D. S., M. D.
Acting Dean, Box 834.

New Orleans, May 21, 1872.

May '72.

### OHIO COLLEGE
—OF—
### Dental Surgery.

The Twenty-seventh Annual Session of the Ohio College of Denta Surgery, will commence on Tuesday, the 15th of October next, and continue until March 1st, 1873.

### FACULTY:

JAMES TAYLOR, M. D., D. D. S.,
Emeritus Professor of Institutes of Dental Science.
C. KEARNS, M. D.,
Professor of Anatomy.
J. S. CASSIDY, D. D. S.,
Professor of Chemistry and Therapeutics.
EDWARD RIVES, M. D.,
Professor of Physiology and Histology.
F. BRUNNING, M. D.,
Professor of Pathology and Therapeutics.
J. TAFT, D. D. S.,
Professor of Operative Dentistry and Dental Hygiene.
C. PALMER, D. D. S.,
Professor of Mechanical Dentistry and Mettalurgy.
WILL TAFT, D. D. S.,
Professor of Clinical Dentistry.
A. SCHWAGMEYER, M. D.,
Demonstrator of Anatomy.

### TEXT BOOKS:

Gray's and Wilson's Anatomy; Dalton's or Carpenter's Physiology; Williams' Pathology; Fown's, Turner's, Graham's, or Brandt and Taylor's Chemistry; Taft's Operative Dentistry; Richardson's Mechanical Dentistry; Harris' Principles and Practtce of Dental Science, Tomes Dental Surgery, Watts' Chemical Essays, Harris' Dental Dictionary.

### TERMS OF ADMISSION:

Tickets must be procured at the beginning of the session.

| | |
|---|---|
| Tickets for the course, - - - - | $100 00 |
| Demonstrator's Ticket for Anatomy, - - | 5 00 |
| Matriculation Fee, but once, - - - | 5 00 |
| Diploma Fee, - - - - - - | 30 00 |

### TERMS OF GRADUATION:

Two courses, (including one of the dissections,) with a satisfactory dental pupilage.

Eight years reputable practice, or a satisfactory examination upon the branches of the Junior Course in this Institution will be received as equal to one course.

An acceptible thesis upon some subject on Dental science.

Must possess a good moral character, and be twenty-one years old.

For further information, address, J. TAFT, *Dean.*
117 West Fourth Street, Cincinnati.

### NEW ORLEANS DENTAL COLLEGE,

### 158 Canal Street, Corner of Baronne.

**1872-73**............SIXTH ANNUAL SESSION............**1872-73**

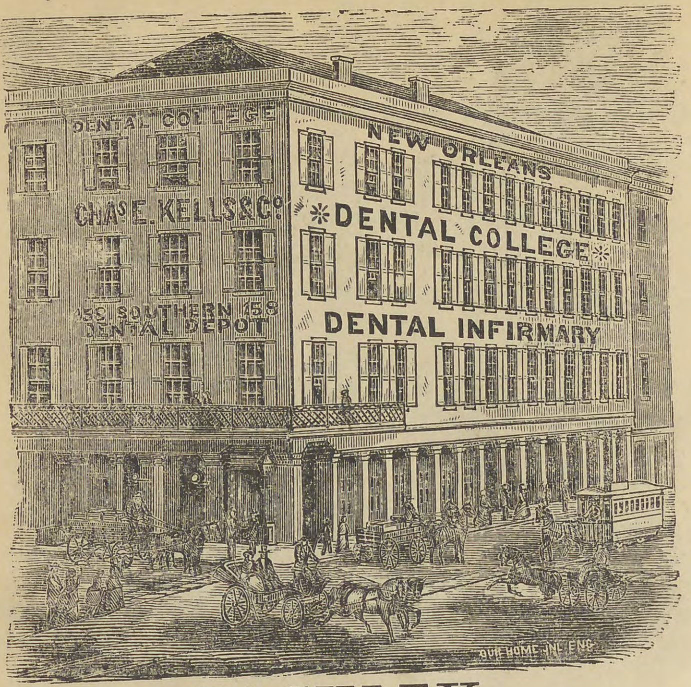

### FACULTY.

—o—

**WM. S. CHANDLER, D. D. S.,** Emeritus Professor of Operative Dental Surgery

---

**JAS. S. KNAPP, D. D. S.,** Professor of Theory and Practice.

**ANDREW F. McLAIN, M. D., D. D. S.,** Professor of Institutes of Medicine and Dentistry and Special Therapeutics.

**CHAS. E. KELLS, D. D. S.,** Professor of the Science of Dental Mechanism.

**S. P. CUTLER, M. D., D. D. S.,** Professor of Chemistry, Mineralogy, Microscopy and Histology.

**JOHN G. ANGELL, D. D. S.,** Professor of Operative Dental Surgery.

**JOHN A. ARRINGTON, D. D. S.,** Professor of Clinical Dentistry and Dental Materia Medica.

**ALFRED W. PERRY, M. D.,** Professor of Anatomy and Physiology.

The **Sixth Regular Course of Lectures** of the NEW ORLEANS DENTAL COLLEGE, for the Session of 1872-73, will commence on the eighteenth of November, and continue four months.

The superior advantages of this location for a Dental College should not be forgotten by Students who live in a colder climate, and who, by coming here, will avoid the severity and rigors of a Northern winter, and enjoy four months of comparatively mild and pleasant weather. To those in delicate health this is an inducement of no inconsiderable importance.

☞ The Faculty is composed entirely, with one exception, of gentlemen in active Dental practice.

| | |
|---|---|
| Professors' Tickets, for a full course.............. | $100 |
| Matriculation Fee, (paid once).............. | 5 |
| Diploma Fee.............. | 30 |

Board and lodging can be procured as cheaply in New Orleans as any large city in the country. For further information, address the Dean of the Faculty,

**JAS. S. KNAPP, D. D. S.,**
Office, No. 15 Baronne street, New Orleans.

Or the Secretary,
Prof. A. F. McLAIN, M. D., D. D. S.,
Acting Dean, Box 834.

### OHIO COLLEGE
—OF—
### Dental Surgery.

The Twenty-seventh Annual Session of the Ohio College of Denta Surgery, will commence on Tuesday, the 15th of October next, and continue until March 1st, 1873.

### FACULTY:

JAMES TAYLOR, M. D., D. D. S.,
Emeritus Professor of Institutes of Dental Science.
C. KEARNS, M. D.,
Professor of Anatomy.
J. S. CASSIDY, D. D. S.,
Professor of Chemistry and Therapeutics.
EDWARD RIVES, M. D.,
Professor of Physiology and Histology.
F. BRUNNING, M. D.,
Professor of Pathology and Therapeutics.
J. TAFT, D. D. S.,
Professor of Operative Dentistry and Dental Hygiene.
C. PALMER, D. D. S.,
Professor of Mechanical Dentistry and Mettalurgy.
WILL TAFT, D. D. S.,
Professor of Clinical Dentistry.
A. SCHWAGMEYER, M. D.,
Demonstrator of Anatomy.

### TEXT BOOKS:

Gray's and Wilson's Anatomy; Dalton's or Carpenter's Physiology; Williams' Pathology; Fown's, Turner's, Graham's, or Brandt and Taylor's Chemistry; Taft's Operative Dentistry; Richardson's Mechanical Dentistry; Harris' Principles and Practtce of Dental Science, Tomes Dental Surgery, Watts' Chemical Essays, Harris' Dental Dictionary.

### TERMS OF ADMISSION:

Tickets must be procured at the beginning of the session.

| | |
|---|---|
| Tickets for the course, - - - - | $100 00 |
| Demonstrator's Ticket for Anatomy, - - | 5 00 |
| Matriculation Fee, but once, - - - | 5 00 |
| Diploma Fee, - - - - - - | 30 00 |

### TERMS OF GRADUATION:

Two courses, (including one of the dissections,) with a satisfactory dental pupilage.

Eight years reputable practice, or a satisfactory examination upon the branches of the Junior Course in this Institution will be received as equal to one course.

An acceptible thesis upon some subject on Dental science.

Must possess a good moral character, and be twenty-one years old.

For further information, address, J. TAFT, *Dean.*
117 West Fourth Street, Cincinnati.

### NEW ORLEANS DENTAL COLLEGE,

**158 Canal Street, Corner of Baronne.**

**1872-73**..............SIXTH ANNUAL SESSION..............**1872-73**

### FACULTY.

---o---

**WM. S. CHANDLER, D. D. S.,** Emeritus Professor of Operative Dental Surgery

---

**JAS. S. KNAPP, D. D. S.,** Professor of Theory and Practice.

**ANDREW F. McLAIN, M. D., D. D. S.,** Professor of Institutes of Medicine and Dentistry and Special Therapeutics.

**CHAS. E. KELLS, D. D. S.,** Professor of the Science of Dental Mechanism.

**S. P. CUTLER, M. D., D. D. S.,** Professor of Chemistry, Mineralogy, Microscopy and Histology.

**JOHN G. ANGELL, D. D. S.,** Professor of Operative Dental Surgery.

**JOHN A. ARRINGTON, D. D. S.,** Professor of Clinical Dentistry and Dental Materia Medica.

**ALFRED W. PERRY, M. D.,** Professor of Anatomy and Physiology.

The **Sixth Regular Course of Lectures** of the NEW ORLEANS DENTAL COLLEGE, for the Session of 1872–73, will commence on the eighteenth of November, and continue four months.

The superior advantages of this location for a Dental College should not be forgotten by Students who live in a colder climate, and who, by coming here, will avoid the severity and rigors of a Northern winter, and enjoy four months of comparatively mild and pleasant weather. To those in delicate health this is an inducement of no inconsiderable importance.

☞ The Faculty is composed entirely, with one exception, of gentlemen in active Dental practice.

| | |
|---|---|
| Professors' Tickets, for a full course.................................... | **$100** |
| Matriculation Fee, (paid once).............................................. | **5** |
| Diploma Fee.................................................................. | **30** |

Board and lodging can be procured as cheaply in New Orleans as any large city in the country. For further information, address the Dean of the Faculty,

**JAS. S. KNAPP, D. D. S.,**
Office, No. **15** Baronne street, New Orleans.

Or the Secretary,
Prof. A. F. McLAIN, M. D., D. D. S.,
Acting Dean, Box **834**.

### OHIO COLLEGE

—OF—

### Dental Surgery.

The Twenty-seventh Annual Session of the Ohio College of Denta Surgery, will commence on Tuesday, the 15th of October next, and continue until March 1st, 1873.

### FACULTY:

JAMES TAYLOR, M. D., D. D. S.,
Emeritus Professor of Institutes of Dental Science.

C. KEARNS, M. D.,
Professor of Anatomy.

J. S. CASSIDY, D. D. S.,
Professor of Chemistry and Therapeutics.

EDWARD RIVES, M. D.,
Professor of Physiology and Histology.

F. BRUNNING, M. D.,
Professor of Pathology and Therapeutics.

J. TAFT, D. D. S.,
Professor of Operative Dentistry and Dental Hygiene.

C. PALMER, D. D. S.,
Professor of Mechanical Dentistry and Mettalurgy.

WILL TAFT, D. D. S.,
Professor of Clinical Dentistry.

A. SCHWAGMEYER, M. D.,
Demonstrator of Anatomy.

### TEXT BOOKS:

Gray's and Wilson's Anatomy; Dalton's or Carpenter's Physiology; Williams' Pathology; Fown's, Turner's, Graham's, or Brandt and Taylor's Chemistry; Taft's Operative Dentistry; Richardson's Mechanical Dentistry; Harris' Principles and Practtce of Dental Science, Tomes Dental Surgery, Watts' Chemical Essays, Harris' Dental Dictionary.

### TERMS OF ADMISSION:

Tickets must be procured at the beginning of the session.

| | |
|---|---|
| Tickets for the course, - - - - | $100 00 |
| Demonstrator's Ticket for Anatomy, - - | 5 00 |
| Matriculation Fee, but once, - - - | 5 00 |
| Diploma Fee, - - - - - - | 30 00 |

### TERMS OF GRADUATION:

Two courses, (including one of the dissections,) with a satisfactory dental pupilage.

Eight years reputable practice, or a satisfactory examination upon the branches of the Junior Course in this Institution will be received as equal to one course.

An acceptible thesis upon some subject on Dental science.

Must possess a good moral character, and be twenty-one years old.

For further information, address, J. TAFT, *Dean.*
117 West Fourth Street, Cincinnati.

### NEW ORLEANS DENTAL COLLEGE,

**158 Canal Street, Corner of Baronne.**

**1872-73**.............SIXTH ANNUAL SESSION.............**1872-73**

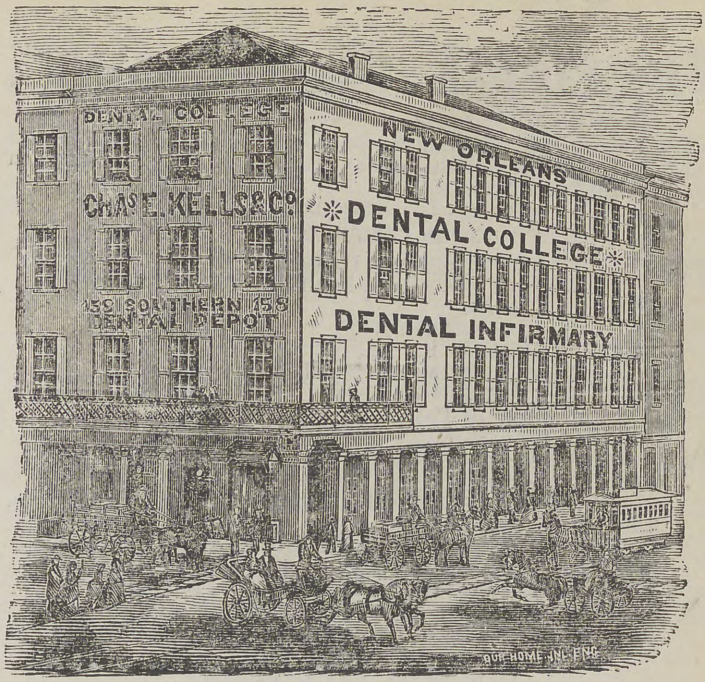

### FACULTY.

**WM. S. CHANDLER, D. D. S.,** Emeritus Professor of Operative Dental Surgery

---

**JAS. S. KNAPP, D. D. S.,** Professor of Theory and Practice.

**ANDREW F. McLAIN, M. D., D. D. S.,** Professor of Institutes of Medicine and Dentistry and Special Therapeutics.

**CHAS. E. KELLS, D. D. S.,** Professor of the Science of Dental Mechanism.

**S. P. CUTLER, M. D., D. D. S.,** Professor of Chemistry, Mineralogy, Microscopy and Histology.

**JOHN G. ANGELL, D. D. S.,** Professor of Operative Dental Surgery.

**JOHN A. ARRINGTON, D. D. S.,** Professor of Clinical Dentistry and Dental Materia Medica.

**ALFRED W. PERRY, M. D.,** Professor of Anatomy and Physiology.

The **Sixth Regular Course of Lectures** of the NEW ORLEANS DENTAL COLLEGE, for the Session of 1872–73, will commence on the eighteenth of November, and continue four months.

The superior advantages of this location for a Dental College should not be forgotten by Students who live in a colder climate, and who, by coming here, will avoid the severity and rigors of a Northern winter, and enjoy four months of comparatively mild and pleasant weather. To those in delicate health this is an inducement of no inconsiderable importance.

☞ The Faculty is composed entirely, with one exception, of gentlemen in active Dental practice.

| | |
|---|---|
| Professors' Tickets, for a full course | **$100** |
| Matriculation Fee, (paid once) | **5** |
| Diploma Fee | **30** |

Board and lodging can be procured as cheaply in New Orleans as any large city in the country. For further information, address the Dean of the Faculty,

**JAS. S. KNAPP, D. D. S.,**
Office, No. **15** Baronne street, New Orleans.

Or the Secretary, Prof. A. F. McLAIN, M. D., D. D. S.,
Acting Dean, Box **834**.

### OHIO COLLEGE

—OF—

### Dental Surgery.

The Twenty-seventh Annual Session of the Ohio College of Dental Surgery, will commence on Tuesday, the 15th of October next, and continue until March 1st, 1873.

### FACULTY:

JAMES TAYLOR, M. D., D. D. S.,
Emeritus Professor of Institutes of Dental Science.
C. KEARNS, M. D.,
Professor of Anatomy.
J. S. CASSIDY, D. D. S.,
Professor of Chemistry and Therapeutics.
EDWARD RIVES, M. D.,
Professor of Physiology and Histology.
F. BRUNNING, M. D.,
Professor of Pathology and Therapeutics.
J. TAFT, D. D. S.,
Professor of Operative Dentistry and Dental Hygiene.
C. PALMER, D. D. S.,
Professor of Mechanical Dentistry and Mettalurgy.
WILL TAFT, D. D. S.,
Professor of Clinical Dentistry.
A. SCHWAGMEYER, M. D.,
Demonstrator of Anatomy.

### TEXT BOOKS

Gray's and Wilson's Anatomy; Dalton's or Carpenter's Physiology; Williams' Pathology; Fown's, Turner's, Graham's, or Brandt and Taylor's Chemistry; Taft's Operative Dentistry; Richardson's Mechanical Dentistry; Harris' Principles and Practce of Dental Science, Tomes Dental Surgery, Watts' Chemical Essays, Harris' Dental Dictionary.

### TERMS OF ADMISSION

Tickets must be procured at the beginning of the session.

| | |
|---|---|
| Tickets for the course, - - - - | $100 00 |
| Demonstrator's Ticket for Anatomy, - - | 5 00 |
| Matriculation Fee, but once, - - - | 5 00 |
| Diploma Fee, - - - - - - | 30 00 |

### TERMS OF GRADUATION:

Two courses, (including one of the dissections,) with a satisfactory dental pupilage.

Eight years reputable practice, or a satisfactory examination upon the branches of the Junior Course in this Institution will be received as equal to one course.

An acceptible thesis upon some subject on Dental science.

Must possess a good moral character, and be twenty-one years old.

For further information, address, J. TAFT, *Dean*.
117 West Fourth Street, Cincinnati.

### NEW ORLEANS DENTAL COLLEGE,

**158 Canal Street, Corner of Baronne.**

**1872-73**............SIXTH ANNUAL SESSION............**1872-73**

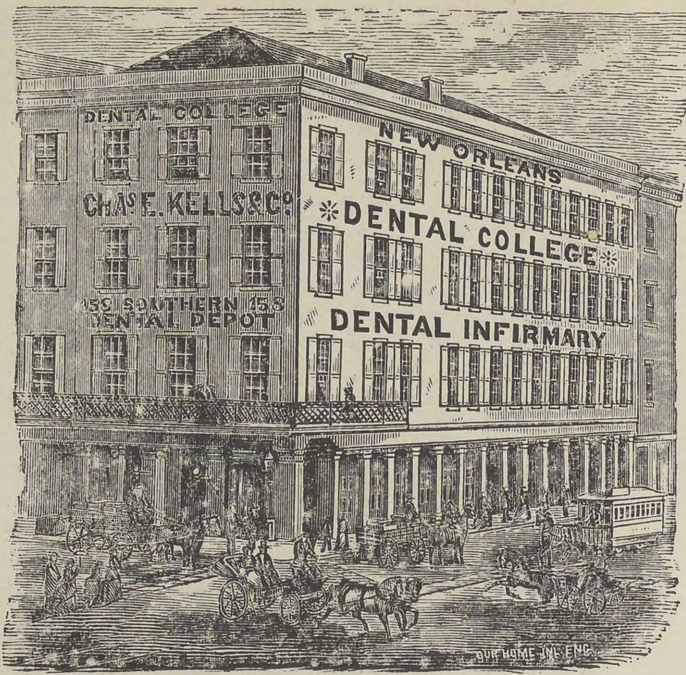

### FACULTY.

**WM. S. CHANDLER, D. D. S.**, Emeritus Professor of Operative Dental Surgery

---

**JAS. S. KNAPP, D. D. S.**, Professor of Theory and Practice.

**ANDREW F. McLAIN, M. D., D. D. S.**, Professor of Institutes of Medicine and Dentistry and Special Therapeutics.

**CHAS. E. KELLS, D. D. S.**, Professor of the Science of Dental Mechanism.

**S. P. CUTLER, M. D., D. D. S.**, Professor of Chemistry, Mineralogy, Microscopy and Histology.

**JOHN G. ANGELL, D. D. S.**, Professor of Operative Dental Surgery.

**JOHN A. ARRINGTON, D. D. S.**, Professor of Clinical Dentistry and Dental Materia Medica.

**ALFRED W. PERRY, M. D.**, Professor of Anatomy and Physiology.

The **Sixth Regular Course of Lectures** of the NEW ORLEANS DENTAL COLLEGE, for the Session of 1872-73, will commence on the eighteenth of November, and continue four months.

The superior advantages of this location for a Dental College should not be forgotten by Students who live in a colder climate, and who, by coming here, will avoid the severity and rigors of a Northern winter, and enjoy four months of comparatively mild and pleasant weather. To those in delicate health this is an inducement of no inconsiderable importance.

☞ The Faculty is composed entirely, with one exception, of gentlemen in active Dental practice.

| | |
|---|---|
| Professors' Tickets, for a full course.................................... | **$100** |
| Matriculation Fee, (paid once)........................................ | **5** |
| Diploma Fee..... ............................................................ | **30** |

Board and lodging can be procured as cheaply in New Orleans as any large city in the country. For further information, address the Dean of the Faculty,

**JAS. S. KNAPP, D. D. S.,**
Office, No. 15 Baronne street, New Orleans.

Or the Secretary,
Prof. A. F. McLAIN, M. D., D. D. S.,
Acting Dean, Box 834.

### OHIO COLLEGE

—OF—

### Dental Surgery.

The Twenty-seventh Annual Session of the Ohio College of Dental Surgery, will commence on Tuesday, the 15th of October next, and continue until March 1st, 1873.

### FACULTY:

JAMES TAYLOR, M. D., D. D. S.,
Emeritus Professor of Institutes of Dental Science.

C. KEARNS, M. D.,
Professor of Anatomy.

J. S. CASSIDY, D. D. S.,
Professor of Chemistry and Therapeutics.

EDWARD RIVES, M. D.,
Professor of Physiology and Histology.

F. BRUNNING, M. D.,
Professor of Pathology and Therapeutics.

J. TAFT, D. D. S.,
Professor of Operative Dentistry and Dental Hygiene.

C. PALMER, D. D. S.,
Professor of Mechanical Dentistry and Mettalurgy.

WILL TAFT, D. D. S,
Professor of Clinical Dentistry.

A. SCHWAGMEYER, M. D.,
Demonstrator of Anatomy.

### TEXT BOOKS

Gray's and Wilson's Anatomy; Dalton's or Carpenter's Physiology; Williams' Pathology; Fown's, Turner's, Graham's, or Brandt and Taylor's Chemistry; Taft's Operative Dentistry; Richardson's Mechanical Dentistry; Harris' Principles and Practtce of Dental Science, Tomes Dental Surgery, Watts' Chemical Essays, Harris' Dental Dictionary.

### TERMS OF ADMISSION

Tickets must be procured at the beginning of the session.

| | |
|---|---|
| Tickets for the course, | $100 00 |
| Demonstrator's Ticket for Anatomy, | 5 00 |
| Matriculation Fee, but once, | 5 00 |
| Diploma Fee, | 30 00 |

### TERMS OF GRADUATION:

Two courses, (including one of the dissections,) with a satisfactory dental pupilage.

Eight years reputable practice, or a satisfactory examination upon the branches of the Junior Course in this Institution will be received as equal to one course.

An acceptible thesis upon some subject on Dental science.

Must possess a good moral character, and be twenty-one years old.

For further information address, J. TAFT, *Dean.*

117 West Fourth Street, Cincinnati

### NEW ORLEANS DENTAL COLLEGE,

**158 Canal Street, Corner of Baronne.**

**1872-73**............SIXTH ANNUAL SESSION............**1872-73**

### FACULTY.

**WM. S. CHANDLER, D. D. S.,** Emeritus Professor of Operative Dental Surgery

**JAS. S. KNAPP, D. D. S.,** Professor of Theory and Practice.

**ANDREW F. McLAIN, M. D., D. D. S.,** Professor of Institutes of Medicine and Dentistry and Special Therapeutics.

**CHAS. E. KELLS, D. D. S.,** Professor of the Science of Dental Mechanism.

**S. P. CUTLER, M. D., D. D. S.,** Professor of Chemistry, Mineralogy, Microscopy and Histology.

**JOHN G. ANGELL, D. D. S.,** Professor of Operative Dental Surgery.

**JOHN A. ARRINGTON, D. D. S.,** Professor of Clinical Dentistry and Dental Materia Medica.

**ALFRED W. PERRY, M. D.,** Professor of Anatomy and Physiology.

The **Sixth Regular Course of Lectures** of the NEW ORLEANS DENTAL COLLEGE, for the Session of 1872-73, will commence on the eighteenth of November, and continue four months.

The superior advantages of this location for a Dental College should not be forgotten by Students who live in a colder climate, and who, by coming here, will avoid the severity and rigors of a Northern winter, and enjoy four months of comparatively mild and pleasant weather. To those in delicate health this is an inducement of no inconsiderable importance.

☞ The Faculty is composed entirely, with one exception, of gentlemen in active Dental practice.

| | |
|---|---|
| Professors' Tickets, for a full course | $100 |
| Matriculation Fee, (paid once) | 5 |
| Diploma Fee | 30 |

Board and lodging can be procured as cheaply in New Orleans as any large city in the country. For further information, address the Dean of the Faculty,

**JAS. S. KNAPP, D. D. S.,**
Office, No. 15 Baronne street, New Orleans.

Or the Secretary, Prof. A. F. McLAIN, M. D., D. D. S.,
Acting Dean, Box 834.

### OHIO COLLEGE

—OF—

### Dental Surgery.

The Twenty-seventh Annual Session of the Ohio College of Dental Surgery, will commence on Tuesday, the 16th of October next, and continue until March 1st, 1873.

### FACULTY:

JAMES TAYLOR, M. D., D. D. S.,
Emeritus Professor of Institutes of Dental Science.

C. KEARNS, M. D.,
Professor of Anatomy.

J. S. CASSIDY, D. D. S.,
Professor of Chemistry and Therapeutics.

EDWARD RIVES, M. D.,
Professor of Physiology and Histology.

F. BRUNNING, M. D.,
Professor of Pathology and Therapeutics.

J. TAFT, D. D. S.,
Professor of Operative Dentistry and Dental Hygiene.

C. PALMER, D. D. S.,
Professor of Clinical and Mechanical Dentistry.

WILL TAFT, D. D. S.,
Professor of Clinical Dentistry.

A. SCHWAGMEYER, M. D.,
Demonstrator of Anatomy.

### TEXT BOOKS

Gray's and Wilson's Anatomy; Dalton's or Carpenter's Physiology; Williams' Pathology; Fown's, Turner's, Graham's, or Brandt and Taylor's Chemistry: Taft's Operative Dentistry; Richardson's Mechanical Dentistry; Harris' Principles and Practce of Dental Science, Tomes Dental Surgery, Watts' Chemical Essays, Harris' Dental Dictionary.

### TERMS OF ADMISSION

Tickets must be procured at the beginning of the session.

| | |
|---|---|
| Tickets for the course, - - - - - | $100 00 |
| Demonstrator's Ticket for Anatomy, - - | 5 00 |
| Matriculation Fee, but once, - - - | 5 00 |
| Diploma Fee, - - - - - - - | 30 00 |

### TERMS OF GRADUATION:

Two courses, (including one of the dissections,) with a satisfactory dental pupilage.

Eight years reputable practice, or a satisfactory examination upon the branches of the Junior Course in this Institution will be received as equal to one course.

An acceptible thesis upon some subject on Dental science.

Must possess a good moral character, and be twenty-one years old.

For further information address, J. TAFT, *Dean.*
117 West Fourth Street, Cincinnati

158 Canal Street, Corner of Baronne.

**1872-73**............SIXTH ANNUAL SESSION............**1872-73**

### FACULTY.

**WM. S. CHANDLER, D. D. S.,** Emeritus Professor of Operative Dental Surgery

---

**JAS. S. KNAPP, D. D. S.,** Professor of Theory and Practice.

**ANDREW F. McLAIN, M. D., D. D. S.,** Professor of Institutes of Medicine and Dentistry and Special Therapeutics.

**CHAS. E. KELLS, D. D. S.,** Professor of the Science of Dental Mechanism.

**S. P. CUTLER, M. D., D. D. S.,** Professor of Chemistry, Mineralogy, Microscopy and Histology.

**JOHN G. ANGELL, D. D. S.,** Professor of Operative Dental Surgery.

**JOHN A. ARRINGTON, D. D. S.,** Professor of Clinical Dentistry and Dental Materia Medica.

**ALFRED W. PERRY, M. D.,** Professor of Anatomy and Physiology.

The **Sixth Regular Course of Lectures** of the NEW ORLEANS DENTAL COLLEGE, for the Session of 1872-73, will commence on the eighteenth of November, and continue four months.

The superior advantages of this location for a Dental College should not be forgotten by Students who live in a colder climate, and who, by coming here, will avoid the severity and rigors of a Northern winter, and enjoy four months of comparatively mild and pleasant weather. To those in delicate health this is an inducement of no inconsiderable importance.

☞ The Faculty is composed entirely, with one exception, of gentlemen in active Dental practice.

| | |
|---|---|
| Professors' Tickets, for a full course | **$100** |
| Matriculation Fee, (paid once) | **5** |
| Diploma Fee | **30** |

Board and lodging can be procured as cheaply in New Orleans as any large city in the country. For further information, address the Dean of the Faculty,

**JAS. S. KNAPP, D. D. S.,**
Office, No. **15** Baronne street, New Orleans.

Or the Secretary.

Prof. A. F. McLAIN, M. D., D. D. S.,
Acting Dean, Box **834**.

### OHIO COLLEGE
—OF—
### Dental Surgery.

The Twenty-seventh Annual Session of the Ohio College of Dental Surgery, will commence on Tuesday, the 16th of October next, and continue until March 1st, 1873.

### FACULTY:

JAMES TAYLOR, M. D., D. D. S.,
Emeritus Professor of Institutes of Dental Science.
C. KEARNS, M. D.,
Professor of Anatomy.
J. S. CASSIDY, D. D. S.,
Professor of Chemistry and Therapeutics.
EDWARD RIVES, M. D.,
Professor of Physiology and Histology.
F. BRUNNING, M. D.,
Professor of Pathology and Therapeutics.
J. TAFT, D. D. S.,
Professor of Operative Dentistry and Dental Hygiene.
C. PALMER, D. D. S.,
Professor of Clinical and Mechanical Dentistry.
WILL TAFT, D. D. S.,
Professor of Clinical Dentistry.
A. SCHWAGMEYER, M. D.,
Demonstrator of Anatomy.

### TEXT BOOKS

Gray's and Wilson's Anatomy; Dalton's or Carpenter's Physiology; Williams' Pathology; Fown's, Turner's, Graham's, or Brandt and Taylor's Chemistry; Taft's Operative Dentistry; Richardson's Mechanical Dentistry; Harris' Principles and Practce of Dental Science, Tomes Dental Surgery, Watts' Chemical Essays, Harris' Dental Dictionary.

### TERMS OF ADMISSION

Tickets must be procured at the beginning of the session.

| | |
|---|---|
| Tickets for the course, - - - - | $100 00 |
| Demonstrator's Ticket for Anatomy, - - | 5 00 |
| Matriculation Fee, but once, - - - | 5 00 |
| Diploma Fee, - - - - - - | 30 00 |

### TERMS OF GRADUATION:

Two courses, (including one of the dissections,) with a satisfactory dental pupilage.

Eight years reputable practice, or a satisfactory examination upon the branches of the Junior Course in this Institution will be received as equal to one course.

An acceptible thesis upon some subject on Dental science.

Must possess a good moral character, and be twenty-one years old.

For further information address, J. TAFT, *Dean*.
117 West Fourth Street, Cincinnati

### NEW ORLEANS DENTAL COLLEGE,

**158 Canal Street, Corner of Baronne.**

**1872-73**..............SIXTH ANNUAL SESSION..............**1872-73**

### FACULTY.

---o---

**WM. S. CHANDLER, D. D. S.,** Emeritus Professor of Operative Dental Surgery

---

**JAS. S. KNAPP, D. D. S.,** Professor of Theory and Practice.

**ANDREW F. McLAIN, M. D., D. D. S.,** Professor of Institutes of Medicine and Dentistry and Special Therapeutics.

**CHAS. E. KELLS, D. D. S.,** Professor of the Science of Dental Mechanism.

**S. P. CUTLER, M. D., D. D. S.,** Professor of Chemistry, Mineralogy, Microscopy and Histology.

**JOHN G. ANGELL, D. D. S.,** Professor of Operative Dental Surgery.

**JOHN A. ARRINGTON, D. D. S.,** Professor of Clinical Dentistry and Dental Materia Medica.

**ALFRED W. PERRY, M. D.,** Professor of Anatomy and Physiology.

The **Sixth Regular Course of Lectures** of the NEW ORLEANS DENTAL COLLEGE, for the Session of 1872-73, will commence on the eighteenth of November, and continue four months.

The superior advantages of this location for a Dental College should not be forgotten by Students who live in a colder climate, and who, by coming here, will avoid the severity and rigors of a Northern winter, and enjoy four months of comparatively mild and pleasant weather. To those in delicate health this is an inducement of no inconsiderable importance.

☞ The Faculty is composed entirely, with one exception, of gentlemen in active Dental practice.

| | |
|---|---|
| Professors' Tickets, for a full course | $100 |
| Matriculation Fee, (paid once) | 5 |
| Diploma Fee | 30 |

Board and lodging can be procured as cheaply in New Orleans as any large city in the country. For further information, address the Dean of the Faculty,

**JAS. S. KNAPP, D. D. S.,**
Office, No. 15 Baronne street, New Orleans.

Or the Secretary.

Prof. A. F. McLAIN, M. D., D. D. S.,
Acting Dean, Box 834.

### OHIO COLLEGE
—OF—
### Dental Surgery.

The Twenty-seventh Annual Session of the Ohio College of Dental Surgery, will commence on Tuesday, the 16th of October next, and continue until March 1st, 1873.

### FACULTY:

JAMES TAYLOR, M. D., D. D. S.,
Emeritus Professor of Institutes of Dental Science.
C. KEARNS, M. D.,
Professor of Anatomy.
J. S. CASSIDY, D. D. S.,
Professor of Chemistry and Therapeutics.
EDWARD RIVES, M. D.,
Professor of Physiology and Histology.
F. BRUNNING, M. D.,
Professor of Pathology and Therapeutics.
J. TAFT, D. D. S.,
Professor of Operative Dentistry and Dental Hygiene.
C. PALMER, D. D. S.,
Professor of Clinical and Mechanical Dentistry.
WILL TAFT, D. D. S.,
Professor of Clinical Dentistry.
A. SCHWAGMEYER, M. D.,
Demonstrator of Anatomy.

### TEXT BOOKS

Gray's and Wilson's Anatomy; Dalton's or Carpenter's Physiology; Williams' Pathology; Fown's, Turner's, Graham's, or Brandt and Taylor's Chemistry; Taft's Operative Dentistry; Richardson's Mechanical Dentistry; Harris' Principles and Practtce of Dental Science, Tomes Dental Surgery, Watts' Chemical Essays, Harris' Dental Dictionary.

### TERMS OF ADMISSION

Tickets must be procured at the beginning of the session.

| | |
|---|---|
| Tickets for the course, - - - - | $100 00 |
| Demonstrator's Ticket for Anatomy, - - | 5 00 |
| Matriculation Fee, but once, - - - | 5 00 |
| Diploma Fee, - - - - - - | 30 00 |

### TERMS OF GRADUATION:

Two courses, (including one of the dissections,) with a satisfactory dental pupilage.

Eight years reputable practice, or a satisfactory examination upon the branches of the Junior Course in this Institution will be received as equal to one course.

An acceptible thesis upon some subject on Dental science.

Must possess a good moral character, and be twenty-one years old.

For further information address, J. TAFT, *Dean*.
117 West Fourth Street, Cincinnati

### 158 Canal Street, Corner of Baronne.

**1872-73**............SIXTH ANNUAL SESSION............**1872-73**

### FACULTY.

**WM. S. CHANDLER, D. D. S.,** Emeritus Professor of Operative Dental Surgery

**JAS. S. KNAPP, D. D. S.,** Professor of Theory and Practice.

**ANDREW F. McLAIN, M. D., D. D. S.,** Professor of Institutes of Medicine and Dentistry and Special Therapeutics.

**CHAS. E. KELLS, D. D. S.,** Professor of the Science of Dental Mechanism.

**S. P. CUTLER, M. D., D. D. S.,** Professor of Chemistry, Mineralogy, Microscopy and Histology.

**JOHN G. ANGELL, D. D. S.,** Professor of Operative Dental Surgery.

**JOHN A. ARRINGTON, D. D. S.,** Professor of Clinical Dentistry and Dental Materia Medica.

**ALFRED W. PERRY, M. D.,** Professor of Anatomy and Physiology.

The **Sixth Regular Course of Lectures** of the NEW ORLEANS DENTAL COLLEGE, for the Session of 1872-73, will commence on the eighteenth of November, and continue four months.

The superior advantages of this location for a Dental College should not be forgotten by Students who live in a colder climate, and who, by coming here, will avoid the severity and rigors of a Northern winter, and enjoy four months of comparatively mild and pleasant weather. To those in delicate health this is an inducement of no inconsiderable importance.

☞ The Faculty is composed entirely, with one exception, of gentlemen in active Dental practice.

| | |
|---|---|
| Professors' Tickets, for a full course | **$100** |
| Matriculation Fee, (paid once) | **5** |
| Diploma Fee | **30** |

Board and lodging can be procured as cheaply in New Orleans as any large city in the country. For further information, address the Dean of the Faculty,

**JAS. S. KNAPP, D. D. S.,**
Office, No. **15** Baronne street, New Orleans.

**Or the Secretary,**
Prof. A. F. McLAIN, M. D., D. D. S.,
Acting Dean, Box **834.**

### OHIO COLLEGE

—OF—

### Dental Surgery.

The Twenty-seventh Annual Session of the Ohio College of Dental Surgery, will commence on Tuesday, the 16th of October next, and continue until March 1st, 1873.

### FACULTY:

JAMES TAYLOR, M. D., D. D. S.,
Emeritus Professor of Institutes of Dental Science.

C. KEARNS, M. D.,
Professor of Anatomy.

J. S. CASSIDY, D. D. S.,
Professor of Chemistry and Therapeutics.

EDWARD RIVES, M. D.,
Professor of Physiology and Histology.

F. BRUNNING, M. D.,
Professor of Pathology and Therapeutics.

J. TAFT, D. D. S.,
Professor of Operative Dentistry and Dental Hygiene.

C. PALMER, D. D. S.,
Professor of Clinical and Mechanical Dentistry.

WILL TAFT, D. D. S.,
Professor of Clinical Dentistry.

A. SCHWAGMEYER, M. D.,
Demonstrator of Anatomy.

### TEXT BOOKS

Gray's and Wilson's Anatomy; Dalton's or Carpenter's Physiology; Williams' Pathology; Fown's, Turner's, Graham's, or Brandt and Taylor's Chemistry; Taft's Operative Dentistry; Richardson's Mechanical Dentistry; Harris' Principles and Practtce of Dental Science, Tomes Dental Surgery, Watts' Chemical Essays, Harris' Dental Dictionary.

### TERMS OF ADMISSION

Tickets must be procured at the beginning of the session.

| | |
|---|---|
| Tickets for the course, - - - - | $100 00 |
| Demonstrator's Ticket for Anatomy, - - | 5 00 |
| Matriculation Fee, but once, - - - | 5 00 |
| Diploma Fee, - - - - - - | 30 00 |

### TERMS OF GRADUATION:

Two courses, (including one of the dissections,) with a satisfactory dental pupilage.

Eight years reputable practice, or a satisfactory examination upon the branches of the Junior Course in this Institution will be received as equal to one course.

An acceptible thesis upon some subject on Dental science.

Must possess a good moral character, and be twenty-one years old.

For further information address, J. TAFT, *Dean.*

117 West Fourth Street, Cincinnati

